# Development and Optimization of the Veterans Affairs’ National Heart Failure Dashboard for Population Health Management

**DOI:** 10.1016/j.cardfail.2023.08.024

**Published:** 2023-09-25

**Authors:** NICHOLAS BROWNELL, CHAD KAY, DAVID PARRA, SHAWN ANDERSON, BRIANA BALLISTER, BRANDON CAVE, JESSICA CONN, SANDESH DEV, STEPHANIE KAISER, JENNIFER ROGERS, ANNA DREW TOULOUPAS, NATALIE VERBOSKY, NARDINE-MARY YASSA, EMILY YOUNG, BOBACK ZIAEIAN

**Affiliations:** 1 Division of Cardiology, David Geffen School of Medicine at University of California, Los Angeles, Los Angeles, CA;; 2 VA Pharmacy Benefits Management Academic Detailing Services, Hines, IL;; 3 Veterans Integrated Service Network 8, Pharmacy Benefits Management, Department of Veterans Affairs, Tampa, FL;; 4 Veterans Affairs, Gainesville, FL;; 5 Center for Medication Safety, VA Pharmacy Benefits Management Services, Hines VA, Hines, IL;; 6 VA West Palm Beach Medical Center, West Palm Beach, FL;; 7 Northern Arizona VA Health Care System, Prescott, AZ;; 8 Southern Arizona VA Health Care System, Tucson, AZ;; 9 Orlando VA Medical Center, Orlando, FL;; 10 Department of Veterans Affairs, Jacksonville, FL;; 11 Louisville VA Medical Center, Louisville, KY;; 12 James A. Haley Veterans’ Hospital, Tampa, FL;; 13 Bay Pines VA Healthcare System, Bay Pines, FL; 14 VA Sierra Pacific Network (VISN 21) Clinical Resource Hub, Palo Alto, CA.

**Keywords:** Learning health system, population health, natural language processing, medical informatics, heart failure, left ventricular ejection fraction

## Abstract

**Background:**

In 2020, the Veterans Affairs (VA) health care system deployed a heart failure (HF) dashboard for use nationally. The initial version was notably imprecise and unreliable for the identification of HF subtypes. We describe the development and subsequent optimization of the VA national HF dashboard.

**Materials and Methods:**

This study describes the stepwise process for improving the accuracy of the VA national HF dashboard, including defining the initial dashboard, improving case definitions, using natural language processing for patient identification, and incorporating an imaging-quality hierarchy model. Optimization further included evaluating whether to require concurrent ICD-codes for inclusion in the dashboard and assessing various imaging modalities for patient characterization.

**Results:**

Through multiple rounds of optimization, the dashboard accuracy (defined as the proportion of true results to the total population) was improved from 54.1% to 89.2% for the identification of HF with reduced ejection fraction (HFrEF) and from 53.9% to 88.0% for the identification of HF with preserved ejection fraction (HFpEF). To align with current guidelines, HF with mildly reduced ejection fraction (HFmrEF) was added to the dashboard output with 88.0% accuracy.

**Conclusions:**

The inclusion of an imaging-quality hierarchy model and natural-language processing algorithm improved the accuracy of the VA national HF dashboard. The revised dashboard informatics algorithm has higher use rates and improved reliability for the health management of the population.

## Brief Lay Summary

The Veterans Affairs (VA) health care system deployed a heart failure (HF) dashboard in 2020 for use nationally, but it was notably imprecise. This study describes the development and optimization of the VA HF dashboard. The project iteratively applied informatics evaluations and implementation to improve the reliability of standardized case definitions and performance measures for patients with HF by using natural language processing and an imaging-quality hierarchy model. The dashboard accuracy improved from 54.1% to 89.2% for the identification of HF with reduced ejection fraction and from 53.9% to 88.0% for the identification of HF with preserved ejection fraction.

## Tweet

Optimization of the VA’s HF dashboard shows improvement in HFrEF identification from 54.1% to 89.2% and in HFpEF identification from 53.9% to 88.0%.

Heart failure (HF) impacts more than 6 million Americans and costs more than US$ 30 billion annually, with projected costs rising to $69.8 billion by 2030.^1,2^ Within the Veterans Affairs (VA) health care system, HF is a leading diagnosis for hospitalization, and age-adjusted HF admission rates are increasing.^3^ Mechanisms to curb costs and morbidity and mortality rates due to HF include improving the use of guideline-directed medical therapy (GDMT) on a national scale. GDMT is a set of evidence-based medications associated with a near 60% reduction in HF admission or cardiovascular death compared to older HF treatments.^4^ Unfortunately, recent studies of the VA health care system suggest that implementation of GDMT use is suboptimal, with use rates as low as 20%–30% for some GDMT medications; these rates are comparable to national and international registry studies of the general population.^5,6^

As a result, there has been an impetus within the VA to improve HF medication uptake by using Learning Health Systems (LHS) principles. The Institute of Medicine defines LHS as systems that generate and apply the best evidence for health-care decisions for every provider and every patient,^7^ and the VA considers itself part of the LHS, committed to optimizing health care delivery.^8,9^ Disease-specific management dashboards can facilitate best practices in a stepwise fashion: timely monitoring of patients’ health data, seamless extraction of these data from the electronic health record (EHR), incorporation with up-to-date best-practice guidelines, and presentation in a digestible format for both clinicians and hospital administration.^7,10–13^ Within the VA, the Academic Detailing Service (ADS) is a team of clinical pharmacists and informaticists responsible for building and managing population dashboards.^11^ The ADS creates and maintains national dashboards for chronic conditions, such as diabetes mellitus, hypertension, chronic obstructive pulmonary disease, and chronic pain.^14^ Although individual VA centers have created dashboards to monitor HF locally, data validation has revealed issues concerning the accurate capture of information pertinent to HF management.^15,16^ The challenge for the ADS was to develop a dashboard for HF that could accurately identify and phenotype persons with HF across the entire national VA system. To do so, the ADS created case definitions related to HF, translated structured and unstructured EHR data so as to reliably identify persons with HF, accurately classified patients by HF clinical phenotype, and created electronic clinical quality measures (eCQMs) that mirror HF performance measures outlined in national guidelines. ADS also continually monitors dashboard output and solicits feedback from end users to optimize and maintain the accuracy of the dashboard. The purpose of this study is to describe the stepwise process of developing and improving the national VA HF dashboard.

## Methods

### Description of Initial HF Dashboard

The initial iteration of the national VA HF Dashboard (Dashboard 1.0) was released in 2020 and was designed to provide end users with a quick summary of their patients with HF. In order to create such a dashboard, ADS needed to use VA’s central data repository (the Corporate Data Warehouse [CDW]), which includes EHR data from 125 distinct relational Veterans Health Information Systems and Technology Architecture (VistA) instances, each with a unique set of EHR systems.^11,12^ Several CDW sources may capture left ventricular ejection fraction (LVEF) data, used for HF phenotypes.^17–19^ The first CDW source is clinical documentation stored in the VA’s nationwide electronic medical record, Computerized Patient Record System (CPRS).^20^ The second is radiology reports that include multiple imaging modalities except echocardiograms. The third is echocardiography reports, which are stored differently across VistA instances. The fourth is the VA Clinical Assessment and Reporting Tracking (CART) program, a clinical analytic tool used to collect patients’ information pertinent to cardiac procedures that includes a clinician’s input of a singular LVEF value and its source. The source could include echocardiography, ventriculography, magnetic resonance imaging, cardiac computed tomography, and nuclear imaging, such as single-photon emission computerized tomography and positron emission tomography.^21^

Because of the various CDW sources used by the VA, LVEF is largely an unstructured field, not extractable by traditional informatics mechanisms. Each VA health care system uses its own local imaging and data-storage applications, so natural language processing (NLP) served as a solution to capture LVEF estimates accurately from the multiple data sources. The NLP dataset is composed of LVEF values derived from patients’ CPRS, radiology reports and echocardiography reports and has been described previously.^22^ To automate the identification and characterization of persons with HF, ADS developed an algorithm to extract directly LVEF estimates from the NLP dataset and to classify accurately patients on the basis of extracted values. The CART program has a requirement for a singular LVEF recording and is the only CDW source with a structured LVEF field; as a result, LVEF could be extracted directly, without the use of NLP. All LVEF information is then stored in a separate database and validated for accuracy.

Dashboard 1.0 used International Classification of Diseases (ICD) codes or an active HF diagnosis in the patient’s problem list to identify veterans with HF. Regardless of source, the lowest LVEF in the past 3 years was used to determine phenotype classification. In cases where no LVEF was available, ICD codes alone were used for classification. Initial user feedback for Dashboard 1.0 noted significant inaccuracies, such as patients being assigned an incorrect phenotype classification. Studies evaluating ICD-10 codes alone to categorize HF show a lack of sensitivity and specificity^23–25^; similar concerns were seen with Dashboard 1.0, which noted that sensitivity was adequate (97.9%) for HF with reduced ejection fraction (HFrEF), but specificity was poor (35.1%). In contrast, for HF with preserved ejection fraction (HFpEF), sensitivity was poor (34.9%) despite adequate specificity (93.7%). Taken together, this meant that Dashboard 1.0 had an accuracy (defined as the ratio of true results [true positives + true negatives] to the total population) of 54.1% for HFrEF and 53.9% for HFpEF. Given this issue, ADS decided to improve the dashboard identification and classification schema for persons with HF.

### Updating Case Definitions

HF clinical phenotypes and stages of HF direct the evidence-based treatments for each subtype of HF.^17–19^ Dashboard 1.0 used HF case definitions based on the most recent American Heart Association (AHA)/American College of Cardiology (ACC) HF guidelines available.^19^ To begin the improvement process, ADS worked with stakeholders to establish updated HF phenotypes for the development of eCQMs: HFrEF, with LVEF ≤ 40%; HF with mildly reduced ejection fraction (HFmrEF), with LVEF 41%–49%; and HFpEF, with LVEF ≥ 50%.^17^ These eCQMs were specific to patients with symptomatic HF and advanced HF (ACC/AHA stages C and D); management of patients at risk for HF (stage A) and patients with pre-HF (stage B) has different evidence-based treatments and thus is outside the scope of this project.

### Hierarchical Algorithm for LVEF Quality and Classification Logic

The Dashboard 1.0 classification model used the lowest LVEF from any source in the past 3 years for phenotype classification. To improve, ADS used a hierarchical algorithmic approach that considered the quality of the data source used to define LVEF. Only patients with LVEF measurements in the past 3 years were classified in the dashboard. LVEF quality was classified as high, medium or low (Table 1). High-quality LVEF included those derived from NLP of echocardiography reports as well as CART-reported LVEF, depending on the original source. Only echocardiography reports with 1 LVEF value were included in the high-quality category. Although invasive ventriculography use has decreased at the VA over time, it remains a widely performed procedure;^26^ invasive ventriculography can overestimate LVEF,^27^ so medium-quality LVEF included a CART-reported LVEF if the original source was ventriculography, as well as NLP extraction from CPRS. If NLP identified an echocardiography report with more than 1 LVEF value, it was included in the medium-quality category, with a logic developed to further clarify LVEF. Low-quality LVEF included those derived from NLP of radiology reports as well as a CART-reported EF if the original source was a nuclear imaging study, which were ultimately excluded due to their inaccuracy.

If a high-quality image was available from within the past year, this image was prioritized to categorize the patient. If a high-quality image was not available from within the past year, the most recent estimate within the past 3 years was used. A phenotype classification was assigned only if recent LVEF values did not conflict with one another (eg, a patient could not be classified as having both HFrEF and HFpEF). As noted, the team identified issues with NLP recording multiple LVEF values from a single study. In this scenario, a logic was developed to exclude values that did not make sense clinically. If an LVEF value was > 75%, the patient was classified as unspecified. If the difference between the high and low LVEF values was ≤ 10%, an average EF was used. If the difference was > 10%, the high value alone was used.

### Improving Dashboard Output

Key performance indicators (KPIs) are metrics used to monitor the progress of a program, and they were aligned with the most recent AHA/ACC HF guidelines.^17^ The KPIs selected for HF management are reported at the individual provider, facility (and selected subdivisions) and network levels, with the national average displayed for comparison. Notable KPIs included in the VA HF dashboard are: percentage of patients who are at high risk, defined as ≥ 2 HF admissions in the past year and receiving cardiology-specialty services; percentage of appropriate patients on each component of GDMT, depending on LVEF; percentage of appropriate patients on target dosages of GDMT; and percentage of patients taking high-risk medications (Table 2).^17,28^

**Fig. 1.**
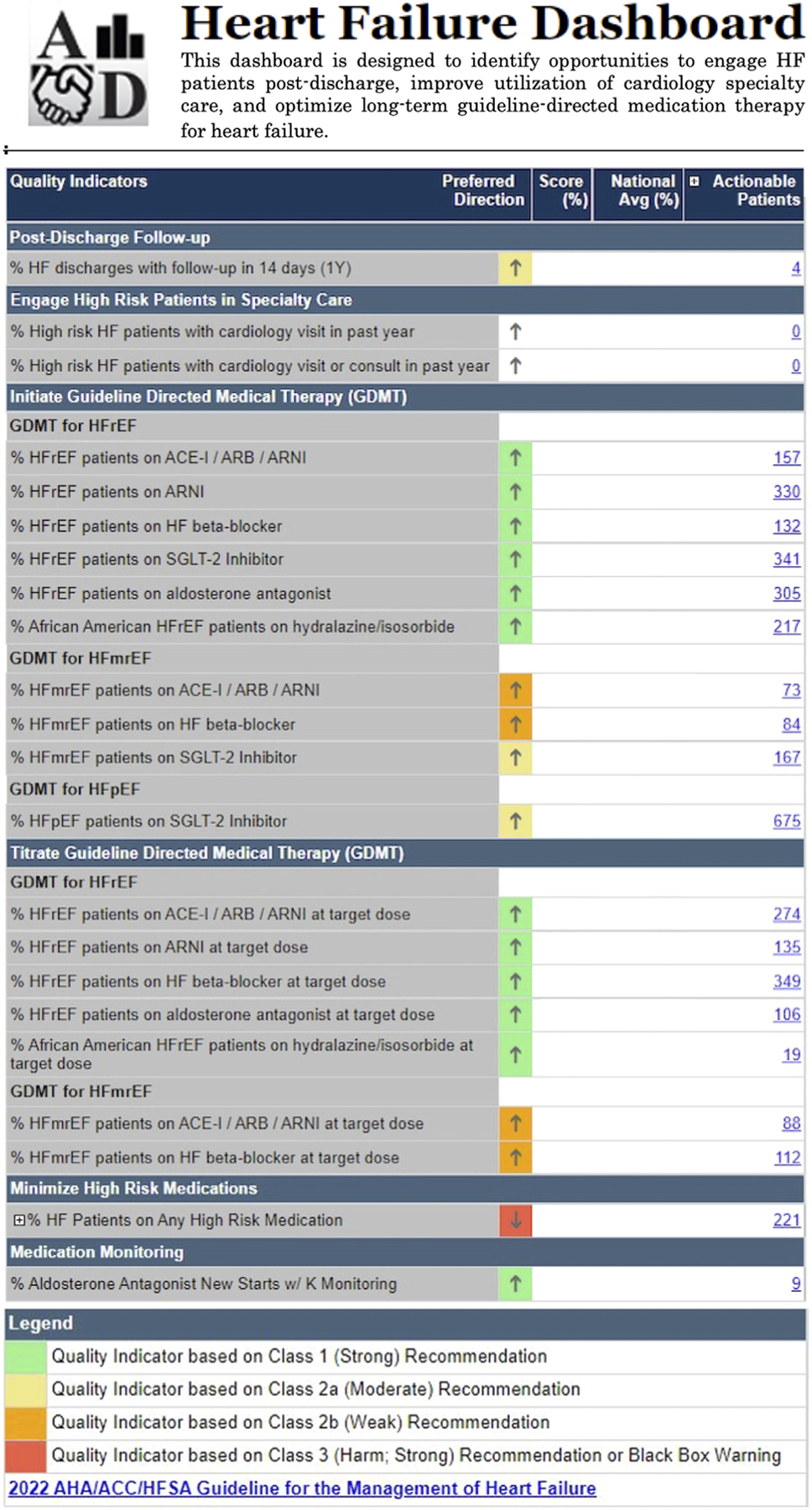
Heart Failure Dashboard 2.0. Key performance indicator (KPI) scores are provided for either individual providers or local facilities. The national average is displayed for comparison. Hyperlinks to actionable patients are presented to facilitate rapid identification of patients in need of appointments, laboratory orders or therapy adjustment.

Based on the user feedback of Dashboard 1.0, the parameters and formatting were updated for a more intuitive interface (Fig. 1). The most recent HF guidelines use a class of recommendation systems to highlight the strength of a recommendation based on the estimated risk/benefit ratio.^17^ To highlight these classes of recommendation, Dashboard 2.0 incorporated a green/yellow/red coloring system to indicate the weight of each recommendation. This was done in concordance with the HF guidelines as well as prior dashboards, where green/yellow/red color coding was universally understood and considered a simple mechanism for communicating risk.^13,17^

Hyperlinks provide immediate visualization of actionable patients. There is also a hyperlink to the updated AHA/ACC HF guidelines for further review of the recommendations pertinent to HF. Every 24 hours, the CDW extracts data pertinent to the KPIs from every VistA instance, such as medication dosaging and frequency, discharge diagnoses, laboratory results, and medical appointments, and stores them in a dataset used to update the eCQM HF dashboard; LVEF data are updated on a quarterly basis.

## Results

The primary hurdle to creating a useful and accurate HF dashboard was ensuring accurate patient identification while considering relevant exclusion criteria. We present the relevant results of the optimization and utilization stages of the Dashboard 2.0 release.

### Optimization: Need for Concurrent ICD Code

Chart review was performed to evaluate patient-inclusion parameters into Dashboard 2.0. Specifically, the team evaluated whether patients who lacked a diagnosis of HF according to ICD codes (or an active HF diagnosis in the problem list) but had reports of an LVEF ≤ 40% met criteria for stage C/D HF.^17^ In the past 3 years, we evaluated 232 patients with reduced LVEFs but without diagnoses of HF by ICD code. The group included 138 patients who had LVEF of 40% and 94 patients who had LVEFs < 40%. Overall, only 18% of these patients were found to have clinical diagnoses of HFrEF in addition to the reduced LVEF. Given this finding, patients without documented ICD diagnoses of HF or active HF diagnoses in the problem list were excluded from the dashboard.

### Optimization: Assessment of Imaging Quality

The NLP of the radiology reports were evaluated for LVEF-derivation accuracy. A total of 181 patients were selected for evaluation. Of these, 154 patients had LVEF results derived from an inaccurate or invalid study (85%), with the majority being nuclear stress tests (n = 130) or noncardiac tests such as gall-bladder ejection fraction imaging (n = 24). Given the marked imprecision of LVEFs derived from NLPs of radiology reports, the decision was made to exclude the entire radiology database from the dashboard. Given the inaccuracy of LVEFs derived from nuclear stress tests, any CART-derived LVEFs that reported “NUCLEAR” as the source were similarly excluded.

### Use: Comparison to Dashboard 1.0

After optimization was performed, a selection of random and nationally representative patients was analyzed to review the overall accuracy of Dashboard 2.0 and to compare it to Dashboard 1.0 (Table 3). The sensitivity and specificity of both the new and old algorithmic decision rules were evaluated and compared to clinician chart reviews. In this cohort of 566 patients, 59 were reported as “unspecified,” meaning their LVEF recordings were discordant or unavailable; the remaining 482 patients underwent classification based on LVEF levels.

Preliminary outcomes included sensitivity, specificity and accuracy for classifying HF. Compared to Dashboard 1.0, overall accuracy for the identification of persons with HFrEF improved from 54.1% to 89.2%. Specificity improved from 35.9% to 91.9%, with a slight decrease in sensitivity from 97.9% to 83.0%. For HFpEF, accuracy improved from 53.9% to 88.0%. Specificity remained stable at 93.7%, and sensitivity improved from 34.9% to 81.6%. In sum, Dashboard 1.0 overidentified persons with HFrEF while underreporting persons with HFpEF, whereas Dashboard 2.0 improved accuracy for the HFrEF and HFpEF phenotypes. Furthermore, the updated case definitions allowed for the novel inclusion of persons with HFmrEF. For these patients, Dashboard 2.0 had an 88.0% accuracy, with 84.1% sensitivity and 89.1% specificity. Given this improvement, Dashboard 2.0 was implemented for use nationally.

### Use: Current Metrics

**Fig. 2.**
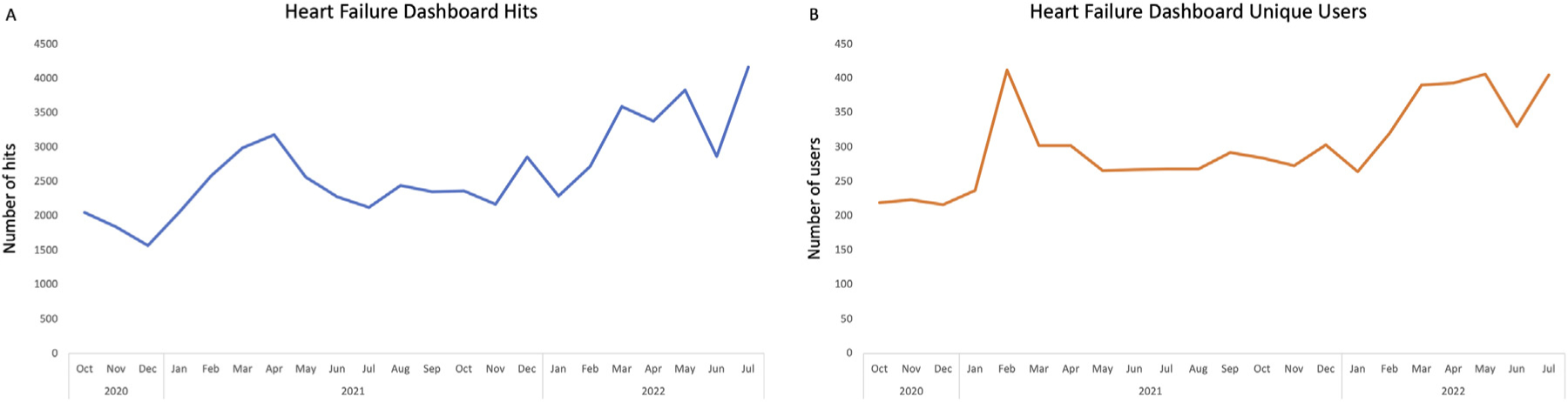
Use of the national dashboard (A) and number of unique users (B).

Current performance in quality indicators is readily available, and an example dashboard is shown (Fig. 1). KPIs are shown in a tabular format, followed by a score, usually the percentage of persons with HF who meet the quality indicator. Nationally, Dashboard 2.0 has been well received. As of July 2022, use is increasing, with 4165 hits per month (Fig. 2A) and 405 unique users per month (Fig. 2B).

## Discussion

Population-management tools such as clinical dashboards allow for timely monitoring of eCQMs on individual and system levels. Creating a panel dashboard that uses NLP for monitoring and promoting best practices within a health care system is a multistep process and warrants ongoing improvement after initial deployment. We report on the development and optimization of the national VA HF dashboard. The VA system is unique, given the numerous EHR sources used regularly for clinical care. When creating a dashboard that uses different EHR databases on a national scale, NLP serves as a viable solution to gather data pertinent to HF care. However, the use of NLP alone did not provide sufficient accuracy for the initial VA HF dashboard. The stepwise process of using ICD codes and chart diagnoses for identifying persons with HF, followed by an indiscriminate approach to obtaining LVEF and HF phenotypes from NLP, led to an inaccurate identification schema and dashboard output; revision of the dashboard needed to account for imaging quality through the creation of an imaging-hierarchy model.

To the best of our knowledge, this is the first report describing the creation and optimization of an HF dashboard for use on a national scale. Prior studies have been conducted at single-center sites, and they highlighted the need for continuous refinement and optimization of HF dashboards to improve performance and use.^15^ We build on the prior literature by describing dashboard creation on a significantly larger scale, including informatics techniques that can improve accuracy and, subsequently, dashboard use. Ultimately, key steps in improving the HF dashboard included ensuring that case definitions were up-to-date concerning revised HF guidelines and creating an imaging hierarchy with concurrent logic to determine LVEF. The use of both an imaging-quality hierarchy model and NLP dramatically improved the accuracy of the HF dashboard at the VA, reaching 88% or higher for both HFrEF and HFpEF. The positive predictive value (PPV) improved dramatically for HFrEF, from 40% to 82%, whereas the PPV for HFpEF remained above 90%. Furthermore, the new inclusion of HFmrEF, in concordance with updated AHA/ACC guidelines, is an example of how population-management tools such as clinical dashboards can and should be continually monitored and updated to ensure that the data presented stay current.

### Limitations

Although current metrics suggest that Dashboard 2.0 has been well received, whether clinician use will translate to meaningful patient impact is yet to be seen. Clinical decision-support tools such as dashboards have generally been associated with positive outcomes^13^; subsequent studies will have to evaluate whether the optimization of the VA HF dashboard has improved GDMT receipt for veterans with HF.

## Conclusion

This study of the VA HF dashboard provides a launchpad and roadmap for other health care networks so they can develop clinical dashboards for HF and other chronic diseases. In conclusion, we propose a 3-step process toward developing and improving a clinical dashboard. The entire process is essential to creating a functional and useful panel dashboard. To begin, we recommend the following:

1. Determine the goal of the dashboard. This will guide case definitions for optimal patient selection and KPIs to gauge performance in terms of optimal clinical practice. Involvement of multidisciplinary stakeholders, such as clinicians, pharmacists and informaticists, can ensure that as practice evolves, case definitions and KPIs will be updated to remain consistent with current best practices. For the HF dashboard, this involved ensuring that case definitions were in concordance with national HF guidelines.
2. Create the necessary search queries and software to extract appropriate patient information from the EHR and present the data in a functional and intuitive manner. When initial methods lead to inaccuracies, revision may involve novel approaches, such as inclusion/exclusion criteria and hierarchical-quality algorithms to ultimately ensure dashboard accuracy.
3. Perform quality control to assess the performance of the dashboard, gauging both the accuracy of patient identification and the usability of the dashboard for end-users. Although it involves substantial manpower, manual chart review ensures that the dashboard output is concordant with the EHR. This step will create a feedback loop such that the dashboard continues to be updated and improved as usage increases.

## Figures and Tables

**Table 1.** Hierarchy of imaging-study quality

	NLP if original source is:	CART if original source is:
High quality	TTE or TEE (1 LVEF reported)	TTE or TEE MRI CT
Medium quality	TTE or TEE (>1 LVEF reported) Clinical documentation	Ventriculography
Low quality (excluded)	Radiology	SPECT PET

CART, Clinical Assessment and Reporting Tracking program; CT, cardiac computed tomography; LVEF, left ventricular ejection fraction; MRI, magnetic resonance imaging; NLP, natural language processing; PET, positron emission tomography; SPECT, single-photon emission computerized tomography; TEE, transesophageal echocardiogram; TTE, transthoracic echocardiogram.

**Table 2.** Targeted high-risk medications, depending on subtype of heart failure

Heart Failure (any subtype)	Heart Failure With Reduced Ejection Fraction
Oral nonsteroidal antiinflammatory drugs	Cilostazol
Dipeptidyl peptidase-4 inhibitors	High-risk antiarrhythmics
Alogliptin	Disopyramide
Saxagliptin	Dronedarone
Thiazolidinediones	Flecainide
Pioglitazone	Propafenone
Rosiglitazone	Select calcium channel blockers
	Diltiazem
	Verapamil
	Nifedipine

**Table 3.** Comparison between HF Dashboard 1.0 and 2.0

		1.0	2.0
Definition			
	HFrEF	LVEF ≤40%	LVEF ≤40%
	HFmrEF	None	LVEF 41%−49%
	HFpEF	LVEF >40%	LVEF ≥50%
Business Rules			
	Documented HF?	Yes	Yes
	LVEF used	Lowest in past 3 years	Most recent
	Study used	Any study	High-quality image if within 1 year; otherwise, most recent image
Performance: HFrEF			
	Sensitivity	97.9%	83.0%
	Specificity	35.1%	91.9%
	Accuracy	54.1%	89.2%
	PPV	39.6%	81.9%
	NPV	97.5%	92.5%
Performance: HFmrEF			
	Sensitivity	–	84.1%
	Specificity	–	89.1%
	Accuracy	–	88.0%
	PPV	–	68.7%
	NPV	–	95.2%
Performance: HFpEF			
	Sensitivity	34.9%	81.6%
	Specificity	93.7%	93.7%
	Accuracy	53.9%	88.0%
	PPV	96.7%	92.1%
	NPV	39.6%	85.0%

HF, heart failure; HFmrEF, heart failure with mid-range ejection fraction; HFpEF, heart failure with preserved ejection fraction; HFrEF, heart failure with reduced ejection fraction; LVEF, left ventricular ejection fraction; NPV, negative predictive value; PPV, positive predictive value.

## References

[1] Virani SS, Alonso A, Aparicio HJ, Benjamin EJ, Bitten-court MS, Callaway CW, on behalf of the American Heart Association Council on Epidemiology and Prevention Statistics Committee and Stroke Statistics Subcommittee. Heart Disease and Stroke Statistics: 2021 Update: a report from the American Heart Association. Circulation 2021;143. [cited Feb 21, 2022]Available at https://www.ahajournals.org/doi/10.1161/CIR.0000000000000950.

[2] Heidenreich PA, Albert NM, Allen LA, Bluemke DA, Butler J, Fonarow GC, Forecasting the impact of heart failure in the United States: a policy statement from the American Heart Association. Circ: Heart Fail 2013;6:606–19. 23616602 10.1161/HHF.0b013e318291329a PMC3908895

[3] Krishnamurthi N, Francis J, Fihn SD, Meyer CS, Whooley MA. Leading causes of cardiovascular hospitalization in 8.45 million US veterans. PLoS ONE 2018;13:e0193996. 10.1371/journal.pone.0193996 PMC5864414 29566396

[4] Vaduganathan M, Claggett BL, Jhund PS, Cunningham JW, Pedro Ferreira J, Zannad F, Estimating lifetime benefits of comprehensive disease-modifying pharmacological therapies in patients with heart failure with reduced ejection fraction: a comparative analysis of three randomised controlled trials. Lancet 2020;396:121–8. 32446323 10.1016/S0140-6736(20)30748-0

[5] Sandhu AT, Kohsaka S, Turakhia MP, Lewis EF, Heidenreich PA. Evaluation of quality of care for US veterans with recent-onset heart failure with reduced ejection fraction. JAMA Cardiol 2022;7:130. 34757380 10.1001/jamacardio.2021.4585 PMC8581797

[6] Brownell NK, Ziaeian B, Fonarow GC. The gap to fill: rationale for rapid initiation and optimal titration of comprehensive disease-modifying medical therapy for heart failure with reduced ejection fraction. Card Fail Rev 2021;7:e18. 34950508 10.15420/cfr.2021.18 PMC8674626

[7] The Learning Healthcare System: Workshop Summary (IOM Roundtable on Evidence-Based Medicine). Washington, D.C.: National Academies Press; 2007.. [Cited May 9, 2022]. Available at: http://www.nap.edu/cata-log/11903. 21452449

[8] Atkins D, Kilbourne AM, Shulkin D. Moving from discovery to system-wide change: the role of research in a learning health care system: experience from three decades of health systems research in the Veterans Health Administration. Annu Rev Pub Health 2017;38:467–87. 28125386 10.1146/annurev-publhealth-031816-044255

[9] Kilbourne AM, Jones PL, Atkins D. Accelerating implementation of research in Learning Health Systems: lessons learned from VA Health Services Research and NCATS Clinical Science Translation Award programs. J Clin Transl Sci 2020;4:195–200. 32695488 10.1017/cts.2020.25 PMC7348004

[10] McGinnis JM, Fineberg HV, Dzau VJ. Advancing the Learning Health System. N Engl J Med 2021;385:1–5. 34192452 10.1056/NEJMp2103872

[11] Lau MK, Bounthavong M, Kay CL, Harvey MA, Christopher MLD. Clinical dashboard development and use for academic detailing in the U.S. Department of Veterans Affairs. J Am Pharm Assoc 2019;59. S96–103.e3. 10.1016/j.japh.2018.12.006 30713078

[12] Carmichael JM, Meier J, Robinson A, Taylor J, Higgins DT, Patel S. Leveraging electronic medical record data for population health management in the Veterans Health Administration: successes and lessons learned. Am J Health-Sys Pharm 2017;74:1447–59. 10.2146/ajhp161048 28887346

[13] Dowding D, Randell R, Gardner P, Fitzpatrick G, Dykes P, Favela J, Dashboards for improving patient care: review of the literature. Int J Med Informat 2015;84:87–100. 10.1016/j.ijmedinf.2014.10.001 25453274

[14] Reading Turchioe M, Volodarskiy A, Pathak J, Wright DN, Tcheng JE, Slotwiner D. Systematic review of current natural language processing methods and applications in cardiology. Heart 2022;108:909–16. 34711662 10.1136/heartjnl-2021-319769 PMC9046466

[15] Foster M, Albanese C, Chen Q, Sethares KA, Evans S, Lehmann LS, Heart failure dashboard design and validation to improve care of veterans. Appl Clin Inform 2020;11:153–9. 32102107 10.1055/s-0040-1701257 PMC7043954

[16] US Department of Veterans Affairs. Pharmacy benefits management services: academic detailing services: educational materials. Cited Aug 22, 2022]; Available at: https://www.pbm.va.gov/academicdetailingservice-home.asp

[17] Heidenreich PA, Bozkurt B, Aguilar D, Allen LA, Byun JJ, Colvin MM, 2022 AHA/ACC/HFSA Guideline for the Management of Heart Failure: a report of the American College of Cardiology/American Heart Association Joint Committee on Clinical Practice Guidelines. Circulation 2022;145. [Cited May 9, 2022] Available at: https://www.ahajournals.org/doi/10.1161/CIR.0000000000001063. 10.1161/CIR.0000000000001063 35363499

[18] Maddox TM, Januzzi JL, Allen LA, Breathett K, Butler J, Davis LL, 2021 update to the 2017 ACC Expert Consensus Decision Pathway for Optimization of Heart Failure Treatment: answers to 10 pivotal issues about heart failure with reduced ejection fraction. J Am Coll Cardiol 2021;77:772–810. 33446410 10.1016/j.jacc.2020.11.022

[19] Yancy CW, Jessup M, Bozkurt B, Butler J, Casey DE, Drazner MH, American College of Cardiology Foundation, American Heart Association Task Force on Practice Guidelines. 2013 ACCF/AHA guideline for the management of heart failure: a report of the American College of Cardiology Foundation/American Heart Association Task Force on Practice Guidelines. J Am Coll Cardiol 2013;62:e147–239. 23747642 10.1016/j.jacc.2013.05.019

[20] Office of Information & Technology. Computerized Patient Record System (CPRS) User Guide: GUI version. 2022. Available at: https://www.va.gov/vdl/documents/Clinical/Comp_Patient_Recrd_Sys_(CPRS)/cprsguium.pdf

[21] US Department of Veterans Affairs. Quality and patient safety: the Clinical Assessment Reporting and Tracking (CART) Program. 2021 Cited Aug 22, 2022. Available at https://www.va.gov/QUALITYANDPA-TIENTSAFETY/cart/index.asp

[22] Patterson OV, Freiberg MS, Skanderson MJ, Fodeh S, Brandt CA, DuVall SL. Unlocking echocardiogram measurements for heart disease research through natural language processing. BMC Cardiovasc Disord 2017;17:151. 28606104 10.1186/s12872-017-0580-8 PMC5469017

[23] Bosco-Lévy P, Duret S, Picard F, Dos Santos P, Puymirat E, Gilleron V, Diagnostic accuracy of the *International Classification of Diseases, Tenth Revision*, codes of heart failure in an administrative database. Pharmacoepidemiol Drug Saf 2019;28:194–200. 30395375 10.1002/pds.4690

[24] Khand AU, Shaw M, Gemmel I, Cleland JGF. Do discharge codes underestimate hospitalisation due to heart failure? Validation study of hospital discharge coding for heart failure. Eur J Heart Fail 2005;7:792–7. 16054867 10.1016/j.ejheart.2005.04.001

[25] Ingelsson E, Ärnlöv J, Sundström J, Lind L. The validity of a diagnosis of heart failure in a hospital discharge register. Eur J Heart Fail 2005;7:787–91. 15916919 10.1016/j.ejheart.2004.12.007

[26] Heidenreich PA, Lin S, Knowles JW, Perez M, Maddox TM, Ho MP, Variation in use of left ventriculography in the Veterans Affairs Health Care System. Circ: Cardiovasc Qual Outcom 2013;6:687–93. 10.1161/CIRCOUTCOMES.113.000199 24192569

[27] Greupner J, Zimmermann E, Grohmann A, Dübel H-P, Althoff T, Borges AC, Head-to-head comparison of left ventricular function assessment with 64-row computed tomography, biplane left cineventriculography, and both 2- and 3-dimensional transthoracic echocardiography. J Am Coll Cardiol 2012;59:1897–907. 22595410 10.1016/j.jacc.2012.01.046

[28] Page RL, O’Bryant CL, Cheng D, Dow TJ, Ky B, Stein CM, Drugs that may cause or exacerbate heart failure: a scientific statement from the American Heart Association. Circulation 2016;134. Cited Jan 11, 2023]Available at: https://www.ahajournals.org/doi/10.1161/CIR.0000000000000426. 10.1161/CIR.0000000000000426 27400984

